# Tropical storms influence the movement behavior of a demersal oceanic fish species

**DOI:** 10.1038/s41598-018-37527-1

**Published:** 2019-02-06

**Authors:** Nathan M. Bacheler, Kyle W. Shertzer, Robin T. Cheshire, Jamie H. MacMahan

**Affiliations:** 1 Southeast Fisheries Science Center, National Marine Fisheries Service, Beaufort, North Carolina 28516 USA; 2 Department of Oceanography, Naval Postgraduate School, Monterey, California 93943 USA

## Abstract

Extreme weather events strongly influence marine, freshwater, and estuarine ecosystems in myriad ways. We quantified movements of a demersal oceanic fish species (gray triggerfish *Balistes capriscus*; *N* = 30) before, during, and after two hurricanes in 2017 using fine-scale acoustic telemetry at a 37-m deep study site in North Carolina, USA. During storms, gray triggerfish movement and emigration rates were 100% and 2550% higher, respectively, than on days with no storms. We found that increased movement rates were much more strongly correlated with wave orbital velocity (i.e., wave-generated oscillatory flow at the seabed) than either barometric pressure or bottom water temperature, two covariates that have been demonstrated to be important for organisms in shallower water. Higher movement rates during storms were due to increased mobility at night, and emigrations typically occurred at night in the direction of deeper water. Overall, we found significant storm effects on the movement behavior of a demersal fish species in the open ocean, despite our study occurring in deeper water than previous studies that have examined storm effects on animal movement. We conclude that tropical storms are a driving force behind the structure of marine ecosystems, in part by influencing movements of mobile animals.

## Introduction

Tropical storms (e.g., cyclones, hurricanes, typhoons) can strongly perturb and restructure marine ecosystems. Wind and waves from storms can disturb the water column and benthos, break and destroy coral reefs, increase sediment and nutrient levels in estuarine and coastal environments via runoff, and change sea level^1^. Physical changes to habitats and environmental conditions during and after storms influence marine organisms in myriad direct and indirect ways. For instance, tropical storms can cause direct mortality of organisms^2^, but also indirect mortality via lack of dissolved oxygen or increased rates of disease due to degraded water conditions^3^. Tropical storms can also alter the habitat use, spawning behavior, and recruitment patterns of marine species (e.g., ref.^4^), in addition to affecting community dynamics and trophic structure^5–7^.

Movement is an essential, unifying feature of the biology and ecology of marine organisms, influencing habitat selection, foraging behavior, predator deterrence, and mating success^8,9^. Species with limited or no mobility (e.g., corals) are more susceptible to storm effects than mobile species because they are unable to move away from the most severe storm conditions^10^. Yet moving away from storms can be costly even for mobile species in terms of the energy spent to migrate^11^, increased mortality rates due to changes in habitat and community dynamics^12^, and loss of reproductive and foraging opportunities. The disruption of normal movement behaviors by storms may therefore have significant fitness-level impacts on marine organisms.

Most attention on storm-related movement of marine organisms has focused on estuaries. Mobile invertebrates and fishes in estuaries tend to move down-estuary as storms approach or make landfall^12–15^. Several species have been found to respond to specific environmental cues, such as a drop in barometric pressure^13,14,16^ or storm runoff^12,15^. Other estuarine and coastal fish species such as smalltooth sawfish (*Pristis pectinata*) and blacktip reef sharks (*Carcharhinus melanopterus*) appear to be influenced by storms only minimally^16,17^.

For oceanic fishes in deeper water, the evidence for storm-related movement is much sparser. The paucity of studies is almost certainly related to the difficulties of tracking fish movement in the open ocean, and as a consequence, it remains unclear if oceanic fishes are less affected by storms, as might be expected given the depth of their habitats. Movements of reef-associated fishes, for instance, are often inferred from pre- and post-hurricane surveys^18–21^, but these results can be misleading without appropriate controls and continuous monitoring^22^. In contrast, Patterson *et al*.^23^ used conventional tagging of red snapper (*Lutjanus campechanus*) to determine that hurricanes affected the probability of movement and distance traveled; similar results have been found for gray triggerfish (*Balistes capriscus*) in the Gulf of Mexico^24^. In the most direct analysis of storm effects on oceanic fish, Secor *et al*.^25^ determined that destratification of the water column due to a storm caused evacuations of telemetered black sea bass (*Centropristis striata*) from three sites on the continental shelf off Maryland, USA.

**Figure 1:**
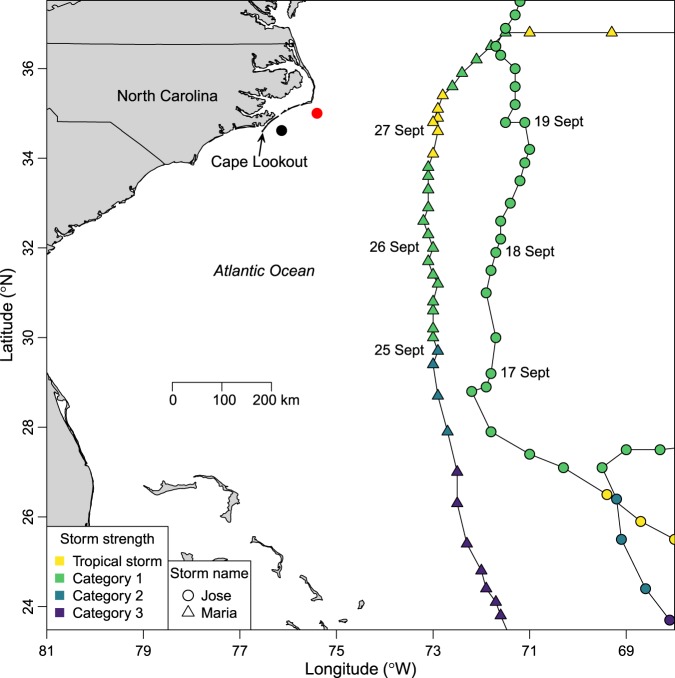
Paths, dates, and intensities of two storms during gray triggerfish (*Balistes capriscus*) tracking on the continental shelf in North Carolina, USA. The 0.48 km^2^ study site is marked with black filled circle (not to scale), NOAA buoy 41025 is marked with red filled circle, storm eyes are indicated by colored circles (Jose) or triangles (Maria), storm strength is indicated by symbol color, and dates of each storm track are also noted when in proximity to the study site.

**Figure 2:**
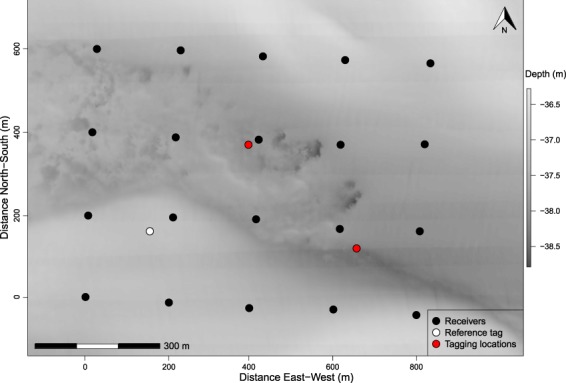
Study site (~34°N, 76°W) where an acoustic positioning system was used to quantify emigration and movement rates of gray triggerfish (*Balistes capriscus*) before, during, and after two hurricanes in North Carolina, 2017. Background image is a multibeam sonar map showing the bathymetry (depth) of the study area; submersible receivers are shown by black filled circles, tagging locations are red filled circles, and the reference tag location is shown by the white filled circle.

Here, we used nearly continuous, fine-scale tracking of gray triggerfish (*Balistes capriscus*) to quantify the ways in which movement behavior was affected by two storms passing near the study area on the continental shelf off North Carolina, USA (Figs 1 and 2). Gray triggerfish are widely distributed in tropical and temperate waters around the western and eastern Atlantic Ocean. They inhabit depths out to approximately 110 m, associate with hard-bottom habitats, and are targeted by recreational and commercial fishers throughout their range^26,27^. Two hurricanes (Jose on 17–19 September 2017 and Maria on 25–27 September 2017) passed near our study area while gray triggerfish were being continuously tracked in 37 m of water (Fig. 1), providing a natural experiment to study the influence of hurricanes on gray triggerfish movement behavior. We examined three potential proximate cues to explain changes in the movement behavior of gray triggerfish during storms: wave orbital velocity (i.e., wave-generated oscillatory flow at the seabed), barometric pressure^13,14^, or bottom temperature^25^. Last, we examined the timing and direction of fish emigrating during and outside of storms. To the best of our knowledge, only one previous study has used fine-scale tracking to examine storm effects on demersal oceanic fish species^25^, and ours is the only one to evaluate multiple potential proximate cues used by oceanic fish to flee from approaching storms.

## Results

**Figure 3:**
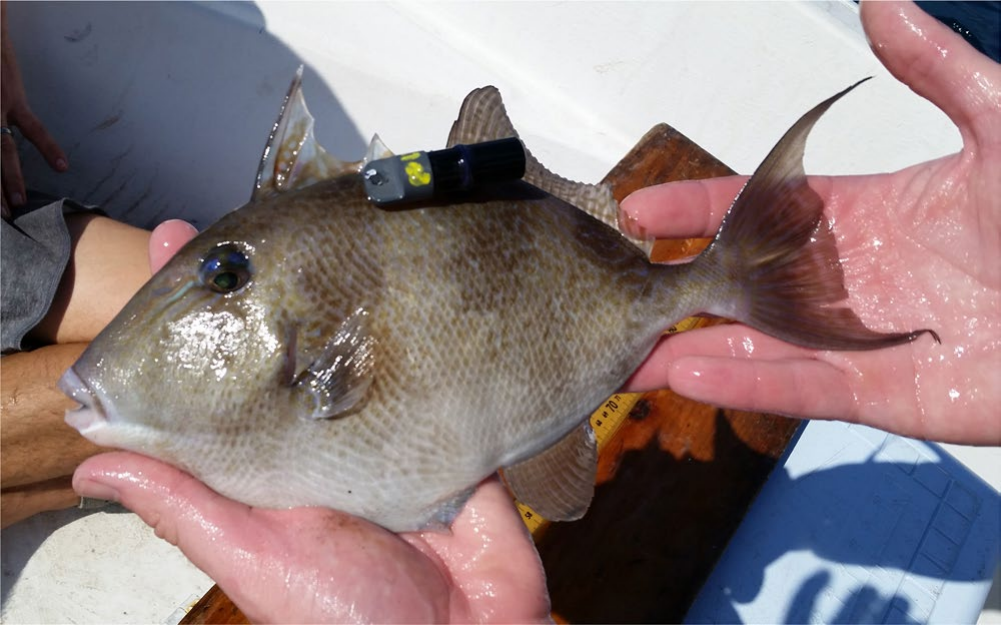
Gray triggerfish (*Balistes capriscus*) outfitted with an externally attached acoustic transmitter on 15 September 2017 in North Carolina, USA.

A total of 30 gray triggerfish were outfitted with transmitters in our study (Fig. 3), ranging from 250 to 335 mm fork length (mean = 291 mm; Table 1). Based on fine-scale movements, we determined that six fish either lost their transmitter or died in the study area, 13 fish permanently emigrated during the study, and 11 fish were alive, retained their tag, and remained in the study area at the end of the study. The six fish that lost their transmitter or died were censored from the analysis starting from when their transmission became stationary, which occurred on days 8, 9, 15, 17, 18, or 26 for the different individuals. Most fish emigrated (temporarily) and returned to the study site at least one time during the study. A total of 104,170 spatial positions were determined for these 30 fish, ranging from 63 to 11,789 positions per fish (mean = 3,472; Table 1).

**Table 1:** Information for individual gray triggerfish (*Balistes capriscus*) outfitted with externally attached transmitters in North Carolina, on 15 September 2017.

Tag	Fork length (mm)	Number of estimated positions	Last day detected	Fate
30	335	1764	27-Sep	Emigrated
31	270	4321	10-Oct	Lost tag or died
32	290	235	29-Sep	Emigrated
33	265	1668	2-Oct	Lost tag or died
34	275	2002	29-Sep	Lost tag or died
35	335	982	1-Oct	Emigrated
36	310	7884	27-Oct	Alive in array
37	280	6992	27-Oct	Alive in array
38	250	8491	27-Oct	Alive in array
39	273	1263	23-Sep	Lost tag or died
40	325	1079	1-Oct	Lost tag or died
41	275	178	18-Sep	Emigrated
42	268	242	15-Oct	Emigrated
43	320	661	26-Sep	Emigrated
44	295	8223	27-Oct	Alive in array
45	312	92	15-Sep	Emigrated
46	285	4345	27-Oct	Alive in array
47	268	8881	27-Oct	Alive in array
48	315	837	22-Sep	Lost tag or died
49	285	5061	27-Oct	Alive in array
50	305	204	18-Sep	Emigrated
51	318	1320	24-Sep	Emigrated
52	275	10912	27-Oct	Alive in array
53	250	167	27-Sep	Emigrated
54	270	9018	27-Oct	Alive in array
55	308	63	16-Sep	Emigrated
56	312	5028	27-Oct	Alive in array
57	305	370	20-Sep	Emigrated
58	255	11789	27-Oct	Alive in array
59	315	98	17-Sep	Emigrated

**Figure 4:**
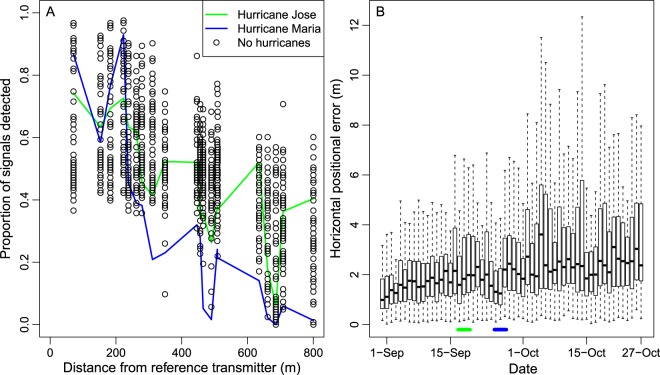
Detection probability and horizontal positional error of a reference transmitter during the telemetry study on the continental shelf of North Carolina, USA. (**A**) Open circles show detection probabilities by distance for each non-hurricane day of the study, and the green and blue lines are the detection probabilities during Hurricanes Jose (green line) and Maria (blue line). (**B**) Horizontal positional error (m) estimated as the distance between the known location of the reference tag deployed in the study area and the various estimated positions for the same reference tag over the course of the 43-d study. The dates of Hurricanes Jose and Maria are indicated in (**B**) by the green and blue horizontal lines, respectively.

Daily detection ranges of the reference transmitter during non-hurricane days were negatively related to the distance between the reference transmitter and receivers (Fig. 4A). Within 300 m, 40–100% of reference transmitter signals each day were detected, but that rate declined to approximately 0 to 50% at 800 m. Hurricane Jose appeared to have a negligible influence on transmitter detections, whereas Hurricane Maria appeared to decrease transmitter detections, particularly at distances greater than 300 m. Median horizontal positional error ranged from about 1 m early in the study to 2–3 m near the end of the study and was unaffected by hurricanes Jose and Maria (Fig. 4B).

**Figure 5:**
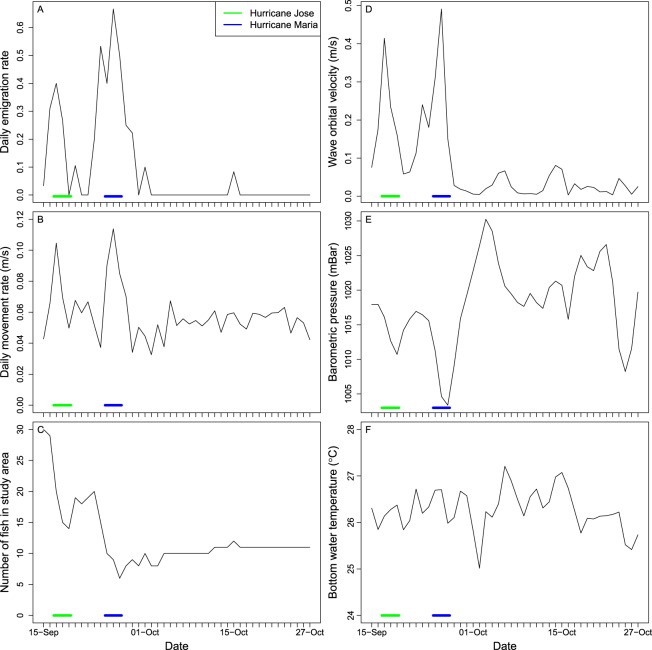
Time series of gray triggerfish *Balistes capriscus* response variables (left column) and predictor variables (right column) during the 43-d acoustic telemetry study in North Carolina, USA, in 2017. Daily means are provided in all plots, and the dates of Hurricanes Jose and Maria are indicated by the green and blue lines, respectively.

Gray triggerfish emigration and movement rates were much higher just before and during hurricanes Jose and Maria than at any other time during the study. Daily emigration rates were very high (>0.3) 1–2 days before and during each storm; this rate can be interpreted as 30% of all fish in the study area as having emigrated on that particular day (Fig. 5A). The highest emigration rate of 0.67 was observed on 26-September (during Hurricane Maria), whereas daily emigration rates were very low (<0.1) when storms were not present (Fig. 5A). Gray triggerfish movement rates followed a similar pattern as emigration rates, being high just before and during storms (>0.08 m/s), but then declining immediately (Hurricane Jose) or within two days (Hurricane Maria) back to rates typical during non-storm days (~ 0.05 m/s; Fig. 5B). Not surprisingly, the number of telemetered gray triggerfish in the study area declined from 30 to 14 during Hurricane Jose and from 20 to 6 fish during Hurricane Maria (Fig. 5C).

Hurricanes Jose and Maria strongly influenced wave orbital velocities and barometric pressure, but not bottom water temperatures, in the study area. Mean daily wave orbital velocity was 0.08 m/s during our study, but increased to over 0.30 m/s during hurricanes Jose and Maria (Fig. 5D). Mean barometric pressure during the study was 1018 mbar, but declined to 1010 mbar during Hurricane Jose and 1002 during Hurricane Maria (Fig. 5E). Bottom water temperature ranged from 25.0 to 27.2 °C during our study, and appeared to be unaffected by the two hurricanes (Fig. 5F).

**Figure 6:**
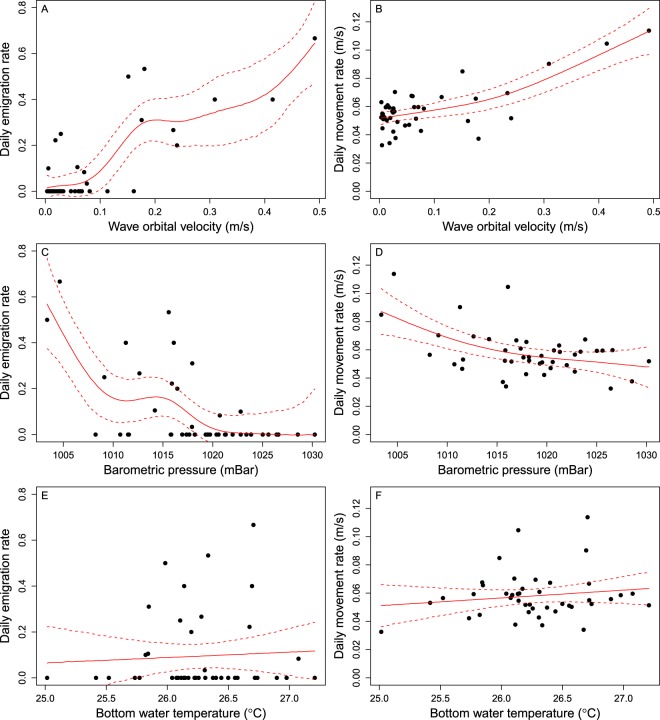
Generalized additive model fits to relationships between the daily emigration rate of gray triggerfish *Balistes capriscus* (left column) or gray triggerfish daily movement rate (m/s; right column) with mean wave orbital velocity (m/s; **A**,**B**), barometric pressure (mbar; **C**,**D**), or bottom water temperature (°C; **E**,**F**) in North Carolina, USA. Raw data are shown by black filled circles, model fits are shown by the solid red line, and dashed red lines are 95% confidence intervals.

Gray triggerfish emigration and movement rates were more highly correlated with wave orbital velocities than either barometric pressure or bottom water temperature. There were strong positive relationships between emigration or movement rates and wave orbital velocities (Fig. 6A,B), and these models explained 72.4 and 59.9% of the model deviance, respectively. The ΔAIC values for models including wave orbital velocities were also lowest of the three possible predictor variables (Table 2). There were negative relationships between emigration or movement rates and barometric pressure (Fig. 6C,D), but these models explained less of the model deviance (50.4 and 29.8%, respectively) and had substantially larger ΔAIC values than wave orbital velocity (Table 2). Bottom water temperature explained very little of the deviance in emigration or movement rates (<3%; Fig. 6E,F) and had the largest ΔAIC values (Table 2).

**Table 2:** Generalized additive model (GAM) results relating gray triggerfish *Balistes capriscus* daily emigration or movement rates in the study area to wave orbital velocity, barometric pressure, or bottom water temperature in North Carolina, 2017.

Response variable	Predictor variable	Dev. expl. (%)	ΔAIC	EDF	*P*
Daily emigration rate	Wave orbital velocity	72.4	0.0	5.0	<0.001
Daily emigration rate	Barometric pressure	50.4	24.8	4.8	<0.001
Daily emigration rate	Bottom temperature	0.4	47.2	1.0	0.70
Daily movement rate	Wave orbital velocity	59.9	0.0	2.1	<0.001
Daily movement rate	Barometric pressure	29.8	24.0	2.1	0.003
Daily movement rate	Bottom temperature	2.2	36.1	1.0	0.34

“Dev expl. (%)” is the deviance explained by the GAM, ΔAIC is the delta Akaike information criterion, EDF is the estimated degrees of freedom of that predictor variable, and *P* is the *P*-value of that predictor variable.

**Figure 7:**
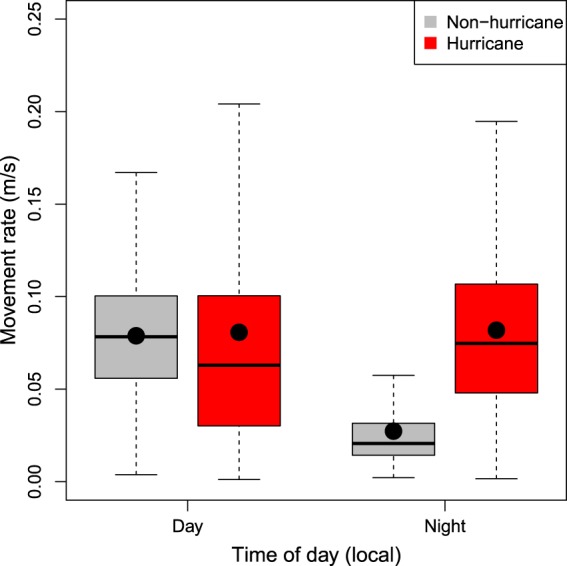
Boxplot of daytime and nighttime movement rates (m/s) of gray triggerfish (*Balistes capriscus*) during (red bars) and outside of times (gray bars) when hurricanes influenced the study area on the continental shelf of North Carolina, USA. Filled black circles show mean values for each group. Daytime was considered 07:00 to 19:00 and night was 19:00 to 07:00 local time.

Gray triggerfish displayed diel differences in movement rates that varied in the presence of hurricanes. On non-hurricane days, mean and median movement rates were approximately 0.08 m/s during the day but declined to around 0.02 m/s during the night, and the time of day effect was retained in the linear model based on AIC (ΔAIC = 23,396 when excluding time of day, *P* < 0.0001; Fig. 7). During hurricanes, however, mean and median movement rates were approximately 0.07 m/s both day and night. Not surprisingly, the hurricane effect was retained in the linear model based on AIC (ΔAIC = 9,100 when excluding the hurricane effect, *P* < 0.0001), as was the interaction between time of day and hurricanes (ΔAIC = 1,133; *P* < 0.0001; Fig. 7).

**Figure 8:**
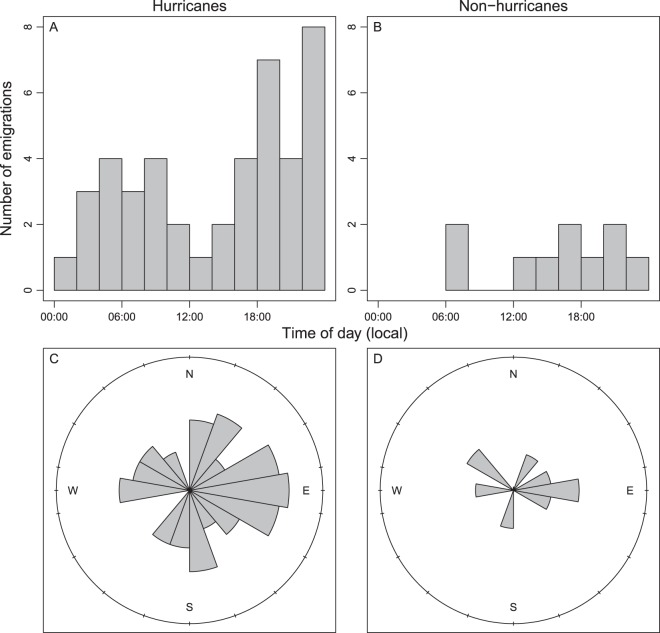
The timing and direction of gray triggerfish (*Balistes capriscus*) emigrations from the study area on the continental shelf of North Carolina, USA, in 2017 during (**A**,**C**) and outside of times (**B**,**D**) when hurricanes influenced the study area. For (**C**) and (**D**), direction of bars indicates direction of emigrations and length of each bar indicates the number of emigrations in that particular direction.

There were 43 emigrations observed during the 6 hurricane days (7.2/day), compared to 10 emigrations observed during 37 non-hurricane days (0.3/day; Fig. 8A,B). Emigrations during hurricane days displayed a significant diel bimodal pattern (χ^2^ = 154, *P* < 0.0001), with modes centered around 06:00 and 20:00 local time (Fig. 8A). Emigrations were lowest midday (~12:00) and in the middle of the night (~00:00). The direction of emigrations during hurricanes was significantly non-random (Rayleigh’s test: $\bar{R}$ = 0.33, *P* = 0.01), occurring primarily eastward towards deeper water (Fig. 8C). During non-hurricane days, emigrations were also diel dependent (χ^2^ = 17.7, *P* = 0.04) and mostly occurred in the afternoon and evening (12:00 to 22:00; Fig. 8B). Emigrations occurring during non-hurricane days were random with respect to direction ($\bar{R}$ = 0.27, *P* = 0.48; Fig. 8D). Given low sample sizes of emigrations occurring outside of storms, there was no statistical difference in emigration direction during and outside of storms (Watson test: *P* > 0.10).

## Discussion

Despite being a demersal fish species in the coastal ocean, gray triggerfish strongly responded to two hurricanes whose eyes passed within 250 km of the study area. Gray triggerfish were much more likely to emigrate from the study area as storms approached, mostly in the direction of deeper water. For fish that remained in the study area, movement rates nearly doubled during storms, and this increase was mostly due to increased movement rates at night, a time when gray triggerfish typically move very little^28^. Differences in gray triggerfish movement rates during and outside of storms could not be attributed to higher positional error rates of transmitters during storms, given spatial precision of 1–3 m each day of the study. Moreover, gray triggerfish appeared to respond to wave orbital velocity as storms approached, and to our knowledge, this study is the first to identify wave orbital velocity as a possible cue used by fishes to detect and escape from storm effects. Overall, we discovered significant storm effects on the movement behavior of a demersal marine fish species, and these effects occurred in much deeper water than has been documented previously for other species.

The nearly universal reaction of marine organisms to storms has been to increase their rate of movement, typically in the direction of deeper water. Jury *et al*.^12^ found higher rates of down-estuary movements of American lobsters (*Homarus americanus*) following a hurricane in 1991 compared to three non-hurricane years, which mirrored the movement patterns of telemetered striped bass (*Morone saxatilis*) in the Hudson River Estuary, New York, after severe storms in 2011^15^. Heupel *et al*.^13^ described the emigration of all (*N* = 13) juvenile blacktip sharks (*Carcharhinus limbatus*) from an estuary into the coastal ocean in the hours before a tropical storm made landfall in Florida. Roberts and Sauer^29^ showed that chokka squid (*Loligo vulgaris reynaudii*) evacuate nearshore spawning grounds for deeper water in South Africa when severe winters increase wave size, resulting in reduced water clarity. Despite our study occurring in deeper water than most of these previous studies, gray triggerfish likewise increased their movement rates and tended to emigrate from the study area towards deeper water. Deeper water likely ameliorates storm effects by providing a buffer against wave energy and perhaps surface noise due to wind, waves, and rain, and it appears to be sought by both estuarine and oceanic species.

Fish use different cues to detect approaching storms. Declining barometric pressure has been most commonly identified as the mechanism by which fish detect storms^13,14,16^. For example, the emigration rates of summer flounder (*Paralichthys dentatus*) from a New Jersey estuary were negatively related to barometric pressure, with fish often emigrating during storm events^14^. Heupel *et al*.^13^ showed that blacktip sharks emigrated as barometric pressure was dropping, despite not having swim bladders that are thought to be used by fish to sense changes in pressure; instead, sharks use vestibular hair cells to detect pressure changes^30^. Other studies have posited that increased storm runoff may have instigated down-estuary movements of organisms^12,15^ and destratification may have caused black sea bass evacuations from sites on the nearshore continental shelf^25^.

We showed that increasing wave orbital velocity at the seabed was the most likely cue used by gray triggerfish to detect approaching storms at a site in the open ocean. Wave orbital velocity explained more of the model deviance and had much lower ΔAIC values than either barometric pressure or bottom water temperature, and these results were consistent for both response variables. Gray triggerfish movement and emigration rates began increasing as the first large, long-period waves arrived at the study area in advance of Hurricanes Jose and Maria, preceding a noticeable decline in barometric pressure; the close temporal match between our response variables and wave orbital velocity was the primary reason it was selected over barometric pressure. Additionally, barometric pressure dropped to a value lower than Hurricane Jose during a late October low pressure system while wave orbital velocity remained low (<0.1 m/s), and the response of gray triggerfish to this low pressure system was negligible. Thus, it appears much more likely that shallow-water organisms sense and respond to barometric pressure (e.g., refs^13,16^) than do oceanic organisms like gray triggerfish. Furthermore, unlike Secor *et al*.^25^, bottom water temperature varied little over the course of our study and explained less than 3% of the variability in emigration and movement rates, suggesting it was not an important cue used by gray triggerfish.

Nearly all studies examining storm effects on fishes are correlational in nature, so ascribing causation is tenuous^31^. While wave orbital velocity correlated strongly with gray triggerfish movements and emigrations, other unmeasured variables could have been correlated with wave orbital velocity and may have been the most important cue used by gray triggerfish. For instance, wave orbital velocity is perfectly correlated with wave dynamic pressure at the seabed, which is the increase and decrease in pressure of a fluid that occelates compared to its static value. During Hurricane Maria, barometric pressure dropped approximately 30 mbar, but the influence of this barometric pressure drop on gray triggerfish at the seabed was smaller than the dynamic pressure range due to water movement caused by large (i.e., 4-m) surface waves. Regardless of the exact cue or cues used by gray triggerfish, surface waves from storms most likely created conditions on the ocean seabed to which gray triggerfish responded.

Emigrations of gray triggerfish from the study area were qualitatively different during storms compared to emigrations that occurred when storms were not present. Gray triggerfish emigration rate was 2550% higher during storms compared to non-hurricane days, consistent with previous studies showing qualitatively different movement behaviors during storms^12,13,15^. Yet in contrast to our hypothesis, increased movement rates during storms were entirely due to higher movement rates at night and most emigrations occurred at night, a time when gray triggerfish typically exhibit decreased movement rates^28^. It is unclear why gray triggerfish move more at night during storms. This nighttime mobility may be intentional and offer a selective advantage (e.g., reduced predation) or it may be due to becoming entrained in strong, storm-induced bottom currents while resting at night.

The time it takes marine habitats and organisms to recover after storms can vary dramatically. Estuaries naturally flush quickly, so it is not surprising that storms tend to have relatively short-lived effects (i.e., weeks to months) on estuarine organisms^6^. For instance, all juvenile blacktip sharks tracked by Heupel *et al*.^13^ returned to their Florida estuary between 5 and 13 d after the storm passed and resumed movement patterns similar to those observed before the storm, suggesting short-lived storm impacts. In contrast, Bell and Hall^21^ documented the ways in which a hurricane damaged artificial reef habitats on the continental shelf in South Carolina: the storm redistributed reef materials, broke apart shipwrecks, and buried other structures. Secor *et al*.^25^ showed that many black sea bass permanently evacuated continental shelf sites after a storm, and those that remained displayed long-term depressed activity levels. In our study, most of the gray triggerfish that emigrated during storms returned within a week after the storms had passed and immediately resumed what appeared to be normal movements. However, many telemetered fish never returned during the study, suggesting that high rates of emigration during storms may permanently redistribute gray triggerfish.

The telemetry system used in our study, which provided fine-scale and highly precise spatial and temporal data, is well suited to elucidate the normally hidden behaviors of demersal oceanic fish species. Highly precise spatial positions were provided every 2–4 min during and outside of hurricanes, which allowed us to quantify in unprecedented detail the movement behaviors of gray triggerfish in relation to two tropical storms. The slight decrease in spatial precision throughout our 43-d study, from approximately 1 m early in the study to 2–3 m late in the study, was likely due to biofouling of receivers^32^.

There were three primary limitations of our study. First, we did not have continuous locations for each telemetered fish in our study; instead, locations were only available when a transmitter’s acoustic signal was detected (i.e., every 2–4 min). It was not possible to determine gray triggerfish movement behaviors in the temporal gap between detections. This is a downside of most tracking systems, but particularly in conventional tagging studies that infer movements from only tagging and recapture locations. Second, it is possible that unusual behaviors due to the tagging process itself confounded storm effects on gray triggerfish movements^33^. We believe this is unlikely given that median movement rates for gray triggerfish were very consistent (i.e., ~ 0.05 m/s) every non-hurricane day regardless of whether it was early or late in the study. Third, the eyes of two storms passed within 250 km of our study area, which elicited obvious responses of gray triggerfish, but both passed by as Category 1 storms; we expect that stronger storms hitting the study area more directly would have much larger effects on gray triggerfish movement patterns.

Tropical storms can be important drivers of terrestrial and aquatic ecosystem structure and function^1,34^. For mobile species, most studies have focused on how storms redistribute terrestrial organisms in space^35,36^, but recent studies including ours have shown that storms similarly influence the behavior and distribution of marine fish species^13–16,25^. Given that extreme weather events such as storms are increasing in magnitude and frequency due to climate change^37,38^, it is likely that such events will exert increasing pressure on estuarine, coastal, and offshore ecosystems. Sustainable management of aquatic species should not overlook the importance of storms in structuring aquatic ecosystems.

## Methods

### Data Collection

Our study took place about 35 km east of Cape Lookout, North Carolina, USA, in 37 m of water. Tagging methods are described in Bacheler *et al*.^39^, so only a brief summary is provided here. We used a Vemco positioning system (VPS^40^) to infer movement behaviors of gray triggerfish; VPS has been used previously to quantify movement rates of various fish species in general and gray triggerfish around artificial reefs specifically^28^. An array of underwater receivers is required in VPS studies, which we deployed on 31 August 2017. We arranged Vemco VR2AR receivers in a 4 × 5 grid with receivers separated 200 m from one another based on previous acoustic detection range estimates for a different species in the region^41^. Thus, our study area was approximately 0.48 km^2^ in size (Fig. 2).

Gray triggerfish (*N* = 30) were outfitted with transmitters on 15 September 2017 (Fig. 3). Fish were captured using traps in the study area, placed in a holding tank, and Vemco V13-1x transmitters were attached externally using polydioxanone absorbable suture material. External attachments were used because detection ranges are longer and the tagging process is much faster than for surgically implanted transmitters^42,43^. Transmitters operated on a frequency of 69 kHz, had a battery life of 904 d, weighed 11 g in air, and had a 110–250 sec ping interval. Receivers were recovered on 27 October 2017, thus fish were tracked for 43 d in total.

We deployed a reference transmitter (Vemco V13T-1×) in the study area (Fig. 2) to quantify water temperature and estimate sound speed, so that fish positions could be estimated precisely. The reference transmitter was also used to estimate transmitter detection range and horizontal positional error. Detection ranges were determined as the proportion of acoustic signals detected by each receiver as a function of the distance between the reference transmitter and each receiver. Horizontal positional error was calculated as the difference in distance between the reference tag’s known location and its estimated VPS position each time it emitted a signal. We developed a boxplot on a daily time step to examine if any changes in horizontal positional error were evident over the course of this study.

### Analyses

First, we determined the fate of each telemetered gray triggerfish by tracking its fine-scale movements through time. We categorized transmitters that stopped moving as “lost tag or died”, transmitters that disappeared at the edge of the study area as “emigrated”, and transmitters that continued to move in the study area at the end of the study as “alive in array”. We only examined the movements of gray triggerfish if they were alive, retained their tag, and were located in the study area.

Second, we tested for the influence of three potential proximate cues that gray triggerfish might use to respond to storms. The first potential cue was barometric pressure, which has been important in previous studies on fishes in shallow, estuarine systems^13,14,16^. Gray triggerfish may be able to sense changes in barometric pressure, despite already being under immense pressure from the water and air at the study site (~4.7 atm). The second was bottom water temperature, which Secor *et al*.^25^ showed increased abruptly during a storm off Maryland, USA, due to destratification of the water column. The last proximate cue we tested was wave orbital velocity at the seabed, which is a measure of the wave-generated oscillatory flow of water that can be calculated from properties of surface wave period (*T*) and height (*H*). Barometric pressure (mbar), wave period, *T* (s), and significant wave height, *H* (m) data were obtained from a nearby (~70 km) NOAA buoy station 41025 (National Data Buoy Center, NOAA) located in 68 m water depth.

The amplitude of the wave orbital velocity at the seabed, *u*_*sb*_ (m/s), was computed as:

1 $${u}_{sb}=\frac{gHk}{2\sigma \,\cosh (kh)}$$

where *g* is gravitational acceleration, *h* is the local water depth, σ (2π/*T*) is the radian wave frequency, cosh is hyperbolic cosine, and *k* is the radian wave number that is iteratively solved for by the linear wave dispersion relationship:

2 $${\sigma }^{2}=gk\tanh \,(kh)$$

where tanh is hyperbolic tangent^44^. Hourly *u*_*sb*_ (Eq. 1) was computed from *H* at the buoy location shoaled to a water depth of 37 m of the field site by

3 $${H}_{h=37m}={H}_{h=68m}\sqrt{\frac{C{g}_{h=68m}}{C{g}_{h=37m}}}$$

where *Cg* is referred to as the wave group velocity, defined as

4 $$Cg=nC,$$

5 $$n=\frac{1}{2}[1+\frac{2kh}{\sinh (2kh)}]$$

6 $$C=\frac{g}{\sigma }\,\tanh (kh)$$

where *n* is a factor, *C* is the wave phase speed, and sinh is hyperbolic sine. Hourly values of wave orbital velocity, as well as those of barometric pressure and temperature, were averaged to represent mean daily measures.

We tested for the influence of wave orbital velocity, barometric pressure, and bottom temperature on two metrics of the movement behavior of gray triggerfish. The first was the daily emigration rate of telemetered gray triggerfish from the study area. The daily emigration rate was calculated as the number of gray triggerfish departing from the study area on day *t* divided by the number of fish that were alive and present in the study area at the beginning of day *t*. Fish losing their transmitter or dying in the study (*N* = 6) area were censored from the analyses on the day in which their tag loss or death occurred and all subsequent days of the study. The second response variable was the movement rate of gray triggerfish that were present in the study area. Movement rates (m/s) were calculated separately for each fish within the acoustic array as the distance moved (m) between sequential detections divided by the time between detections (s). Mean daily movement rates across all fish were calculated for each day of the study.

We examined the relationships between response variables (i.e., daily emigration or movement rates of gray triggerfish) and predictor variables (i.e., wave orbital velocity, barometric pressure, bottom water temperature) using generalized additive models (GAMs). These models are nonparametric regressions that can model nonlinear relationships between response and predictor variables^45,46^. We developed a separate GAM for each unique combination (*N* = 6) of response and predictor variables. Since some predictor variables were correlated, they could not be included together in GAMs. Our GAMs were coded as:

7 $$y=\alpha +s(x)+\varepsilon $$

where *y* is the specific response variable, *α* is the model intercept, *x* is one of three predictor variables, *s* is a nonparametric smoothing function (i.e., cubic spline), and *ε* is a Gaussian error distribution.

We used two criteria to arbitrate among the three predictor variables. First, we examined the deviance explained (analogous to a model’s *R*^2^ value) by each predictor variable as a test for the explanatory power of each predictor variable on each response variable. Second, we calculated the Akaike information criterion (AIC) for each model to further compare the influence of each predictor variable on response variables. For model comparison, we present ΔAIC, which is the AIC value of the model of interest minus the AIC of the best model for a given response variable^47^. A ΔAIC value of zero indicates the best model for that particular response variable. All GAMs were coded and analyzed using the mgcv library (version 1.8–17^48^) in R version 3.4.3^49^. All final GAMs met the assumptions of normally distributed errors and constant variance.

Our third analysis tested for the influence of hurricanes on the daytime and nighttime movement rates of gray triggerfish. Given low nighttime movement rates previously documented for gray triggerfish by Herbig and Szedlmayer^28^, we hypothesized that gray triggerfish movement rates would increase during the day and remain low at night. We developed a boxplot of gray triggerfish movement rates showing day and night movement rates during and outside of hurricanes. Since sunrise was at approximately 0700 and sunset was around 1900 local time during our study, daytime was considered 0700 through 1900 and nighttime was considered 1900 through 0700. Using data from NOAA buoy station 41025, six “hurricane days” were identified as having daily mean wave heights larger than 3 m and daily mean wind speeds greater than 10 m/s: 17–19 September (Hurricane Jose) and 25–27 September (Hurricane Maria; Fig. 1). All other days of the study were considered non-hurricane days. We tested for diurnal and hurricane effects using a linear model as:

8 $$y=\alpha +tod+hur+tod\times hur+{\epsilon }$$

where *y* is gray triggerfish movement rate, *α* is the model intercept, *tod* is the time of day effect, *hur* is the hurricane effect, *tod* × *hur* is an interaction between *tod* and *hur*, and *∈* is Gaussian error. The interaction term tested our specific hypothesis that hurricanes would tend to influence movement rates during the day but not at night. The linear model was developed using the “lm” function in base R^48^, and we again used ΔAIC values for model selection.

Last, we quantified characteristics of gray triggerfish emigration events during and outside of hurricanes. For each emigrating gray triggerfish, we recorded the time of day the emigration occurred and the cardinal direction it was moving towards (based on the last two spatial positions) when it was last detected. We hypothesized that, during hurricanes, gray triggerfish would emigrate towards deeper water (eastward) to seek storm relief and emigrations would occur mainly during the day, when visual acuity was maximized. Furthermore, we hypothesized that emigrations occurring outside of storms would be random with respect to direction and would occur during the day, given their diurnal behavior described by Herbig and Szedlmayer^28^. We used the Pearson’s chi-square test for count data to test for diel dependence of emigration rates during and outside of hurricanes, and we used the Rayleigh’s Test of Uniformity to test for directional dependence of emigration. Last, we used a Watson Two-Sample Test of Homogeneity to determine if emigration directions were different during and outside of hurricanes.

### Ethics Statement

All research activities were carried out under a Scientific Research Permit issued to Nathan Bacheler on 10 April 2017 by the U.S. National Marine Fisheries Service, in accordance with the relevant guidelines and regulations on the ethical use of animals as experimental subjects.

## Data Availability

Due to ethical concerns about making the precise locations of gray triggerfish available to the public, data are not published here but are available upon request to the corresponding author (nate.bacheler@noaa.gov).
